# Biallelic *GINS2* variant p.(Arg114Leu) causes Meier-Gorlin syndrome with craniosynostosis

**DOI:** 10.1136/jmedgenet-2020-107572

**Published:** 2021-08-05

**Authors:** Maria J Nabais Sá, Kerry A Miller, Mary McQuaid, Nils Koelling, Andrew O M Wilkie, Hugo Wurtele, Arjan P M de Brouwer, Jorge Oliveira

**Affiliations:** 1 Department of Human Genetics, Radboud University Medical Center and Donders Institute for Brain, Cognition and Behaviour, Nijmegen, The Netherlands; 2 Unit for Multidisciplinary Research in Biomedicine, Instituto de Ciências Biomédicas Abel Salazar, Universidade do Porto, Porto, Portugal; 3 Clinical Genetics Group, MRC Weatherall Institute of Molecular Medicine, University of Oxford, Oxford, UK; 4 Maisonneuve-Rosemont Hospital Research Center, Montréal, Québec, Canada; 5 Centre for Predictive and Preventive Genetics (CGPP), Institute for Molecular and Cell Biology (IBMC), Universidade do Porto, Porto, Portugal; 6 UnIGENe, i3S - Instituto de Investigação e Inovação em Saúde, Universidade do Porto, Porto, Portugal

**Keywords:** DNA replication, genetics

## Abstract

**Introduction:**

Replication of the nuclear genome is an essential step for cell division. Pathogenic variants in genes coding for highly conserved components of the DNA replication machinery cause Meier-Gorlin syndrome (MGORS).

**Objective:**

Identification of novel genes associated with MGORS.

**Methods:**

Exome sequencing was performed to investigate the genotype of an individual presenting with prenatal and postnatal growth restriction, a craniofacial gestalt of MGORS and coronal craniosynostosis. The analysis of the candidate variants employed bioinformatic tools, *in silico* structural protein analysis and modelling in budding yeast.

**Results:**

A novel homozygous missense variant NM_016095.2:c.341G>T, p.(Arg114Leu), in *GINS2* was identified. Both non-consanguineous healthy parents carried this variant. Bioinformatic analysis supports its classification as pathogenic. Functional analyses using yeast showed that this variant increases sensitivity to nicotinamide, a compound that interferes with DNA replication processes. The phylogenetically highly conserved residue p.Arg114 localises at the docking site of CDC45 and MCM5 at GINS2. Moreover, the missense change possibly disrupts the effective interaction between the GINS complex and CDC45, which is necessary for the CMG helicase complex (Cdc45/MCM2–7/GINS) to accurately operate. Interestingly, our patient’s phenotype is strikingly similar to the phenotype of patients with *CDC45*-related MGORS, particularly those with craniosynostosis, mild short stature and patellar hypoplasia.

**Conclusion:**

*GINS2* is a new disease-associated gene, expanding the genetic aetiology of MGORS.

## Introduction

Meier-Gorlin syndrome (MGORS) is characterised by a triad of clinical findings consisting of: (1) prenatal and postnatal growth retardation, (2) microtia, and (3) absent or hypoplastic patellae.^1^ This disorder is caused by pathogenic variants in genes coding for evolutionarily conserved components of the replication machinery of the nuclear genome—*ORC1*, *ORC4*, *ORC6*, *CDT1*, *CDC6*, *GMNN*, *CDC45*, *MCM5* and *DONSON*.^2–7^ Six ORC proteins (ORC1–6), Cdc6, Cdt1 and a heterohexamer of MCM proteins (MCM2–7) form a prereplication complex, which is activated by binding of Cdc45 and the heterotetramer GINS (GINS1–4) to MCM2–7.^8^ The resultant preinitiation CMG complex (Cdc45/MCM2–7/GINS) is a DNA helicase that separates the two strands of the DNA double helix at replication origins, subsequently enabling their replication.^8^

The genotype of individuals with MGORS requires at least one allele of genes encoding essential DNA replication factors allowing for some residual activity (hypomorphic variant).^2–6^ Furthermore, less severe phenotypes are often associated with two hypomorphic variants, while more severe phenotypes result from a combination of a hypomorphic and a loss-of-function variant.^1^ Nevertheless, in approximately 20% of individuals no pathogenic variants have been detected.^1^ Here, we describe the first patient with a homozygous disease-causing variant in *GINS2*, a subunit of the preinitiation CMG helicase, presenting with craniosynostosis and fulfilling the clinical diagnosis of MGORS.

## Methods

The family was enrolled with informed consent into the Genetics Basis of Craniofacial Malformations study. The individual’s phenotype was longitudinally and systematically evaluated. Exome capture, sequencing and analysis of DNA extracted from peripheral blood cells of the proband and both parents were carried out as described in online supplemental methods. We analysed the data assuming complete penetrance, allowing for the possibility of either a *de novo* variant (dominant) or biallelic inheritance (recessive). *In silico* protein analysis of CMG structures was performed for mutation prediction over stability and interactions, as detailed in online supplemental methods. One patient variant was further characterised using budding yeast *Saccharomyces cerevisiae*, as described in online supplemental methods, online supplemental table 5, online supplemental table 6.

10.1136/jmedgenet-2020-107572.supp1 Supplementary data

10.1136/jmedgenet-2020-107572.supp8 Supplementary data

10.1136/jmedgenet-2020-107572.supp9 Supplementary data

## Results

A 2-month-old girl was referred for genetic evaluation due to intrauterine growth restriction (IUGR), short stature, microcephaly and facial dysmorphisms. She was the only child of a non-consanguineous healthy Portuguese couple with an unremarkable family history. During pregnancy, IUGR was diagnosed at the 29th week of gestation. Fetal structural abnormalities, infections and teratogens were excluded. She was born at 37^+5^ gestational weeks, by eutocic delivery with Apgar scores 9 and 10, at first and fifth minutes. At birth, her weight was 2260 g (1st centile; −2.2 SD), her length was 47 cm (~10th centile) and her head circumference (HC) was 30.5 cm (<1st centile; −3 SD). She had neonatal jaundice and mild hypotonia. Poor suction and feeding difficulties were noticed and gastro-oesophageal reflux was diagnosed. Newborn metabolic screening was normal. Newborn hearing screening failed, but auditory-evoked potentials at age of 10 days were within the normal electrophysiological limits.

**Figure 1:**
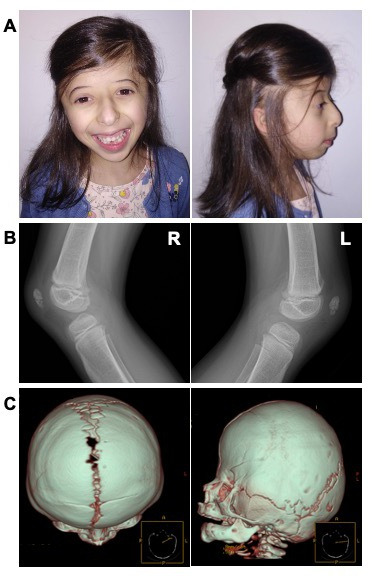
Clinical findings of an individual with a homozygous missense *GINS2* variant. (A) Craniofacial features of Meier-Gorlin syndrome at 6 years of age, including microtia, thin eyebrows, a narrow nose with a convex nasal ridge, microstomia, full lips and microretrognathia. (B) Lateral radiographic view of both knees at 7 years of age, showing hypoplastic patellae. (C) 3D reconstruction of cranial CT scans at 5 months old, demonstrating an incomplete premature fusion of coronal sutures. L, left knee; R, right knee.

She presented with craniofacial dysmorphic features, which evolved with age (figure 1). Her head was microcephalic and brachycephalic and her neck was short. At 6 months, her face was round with a narrow forehead and a low hairline, mid-face hypoplasia and microretrognathia. Ears were small, low set and posteriorly rotated with an atretic external auditory canal. Eyes were prominent and palpebral fissures were downslanted. Her nose was short with a wide, depressed nasal bridge, a convex nasal ridge, hypoplastic nares, low insertion of the columella and long philtrum. Her mouth was small with downturned corners, full lips and a high narrowed palate. She also had short and tapering fingers, short toes, a sacral dimple, an anteriorly placed anus and hypopigmented macules on the abdomen and upper back. At 6 years of age, hypopigmented macules were also observed on the arms and legs.

A bilateral coronal craniosynostosis was confirmed by cranial CT performed at 5 months old (figure 1), and surgically corrected at 17 months. Delayed teeth eruption was observed, with the first tooth erupting after 16 months of age. Her height, weight and HC improved with age (online supplemental table 1). Psychomotor development was adequate. During childhood, she had recurrent respiratory infections.

10.1136/jmedgenet-2020-107572.supp2 Supplementary data

Extensive system-based investigation was performed. Left ureteropelvic ectasia was noticed during an abdominal and renovesical ultrasound at 16 days. During the cardiological examination at 1 month of age, a patent foramen ovale and an atrial septal defect (ASD) of 5 mm with a left-right shunt were diagnosed. At 15 months, no ECG abnormalities were observed. Surgical closure of the ASD took place at 5 years and 9 months old. Four months before this surgery, she had an *ostium secundum*-type ASD of about 10 mm and a sinus venous-type ASD of about 5 mm, resulting in a left-right shunt and dilated right cavities. A mild tricuspid regurgitation with a right ventricle/right auricle gradient of about 16 mm Hg had also been detected. A complete skeletal X-ray at 21 months did not show skeletal abnormalities. Left wrist X-ray at 22 months demonstrated delayed bone age (10–12 months). Ophthalmological evaluation at 22 months diagnosed myopia. Lymphocyte immunophenotyping study at 2 years and 10 months did not show quantitative changes suggestive of any immunodeficiency. Finally, knee radiography, performed at 7 years of age, showed hypoplastic patellae (figure 1).

Given prenatal and postnatal growth delay, bilateral coronal craniosynostosis, cardiac defects and craniofacial dysmorphic features, standard diagnostic genetic investigation was performed. Karyotype, chromosomal microarray and direct sequencing of the *FGFR2*, *FGFR3* (exons 7 and 10) and *TWIST* did not identify pathogenic variants. Through exome sequencing, a homozygous missense variant NM_016095.2:c.341G>T, p.(Arg114Leu), was identified in the *GINS2* gene (MIM*610609) as the most likely candidate genetic cause of the observed primordial dwarfism and craniosynostosis phenotype. Other variants in candidate genes (online supplemental table 2) were excluded from further experimental studies. The single heterozygous variant in *UBQLN3* gene was reported in gnomAD at a low frequency (two heterozygotes listed in this database). As for the compound heterozygous variants identified in four different genes, they were also under-rated considering: (1) the lower deleterious score or inconsistency between bioinformatic predictors (*ANKRD11*, *RIF1* and *SYNJ2*); and (2) the protein’s known biological function did not correlate with the patient’s phenotype (*AHNAK*). The cumulative size of all runs of homozygosity (ROH) was estimated at 18.6 Mb, representing <1% of the genome and excluding any close consanguineous relationship between the parents. The inspection of ROH showed that the *GINS2* candidate variant was located in the patient’s largest ROH (2.04 Mb) detected through exome sequencing data; only four other smaller ROHs (varying between 1.17 and 1.66 Mb) were detected. Populational data (gnomAD^9^) demonstrated that this variant has an extremely low frequency (0.020%; 7/34 498 in the ‘Latino’ population), which can be extrapolated using the Hardy-Weinberg equilibrium to a frequency of homozygotes of about 1/9.72×10^7^. Interestingly, the presence of homozygous variants in *GINS2* is extremely rare in gnomAD^9^: so far, only two missense (and no loss-of-function) homozygous variants have been listed. Of note, this is a small gene with an open reading frame of 555 bp. Finally, the substitution of the highly conserved arginine (down to yeast, considering 11 species; online supplemental figure 1) by a leucine, corresponding to a moderate physicochemical difference (Grantham dist: 102 (0–215)), is classified by bioinformatic analysis as likely pathogenic (PolyPhen-2, SIFT, MutationTaster). The Combined Annotation Dependent Depletion (CADD) score was 28.3.^10^

10.1136/jmedgenet-2020-107572.supp3 Supplementary data

10.1136/jmedgenet-2020-107572.supp4 Supplementary data

Protein structural analysis shows that this missense variant affects one residue (p.Arg114) located in an alpha helix domain and in close proximity to CDC45, MCM5 and GINS3 polypeptides (online supplemental figure 2A). Given the availability of its three-dimensional structure, both from humans and *S. cerevisiae*, we analysed the variant’s impact on protein interaction and stability. In different protein structures (or conformations) from *S. cerevisiae,* the corresponding residue—p.Arg142—establishes several hydrogen bonds with other neighbouring amino acids from Psf2 (GINS2) itself but also with Cdc45 (one conserved residue in both species) and Mcm5 (an isofunctionally substituted residue) (online supplemental figure 2B–D). Structure prediction by comparative modelling of protein three-dimensional structures suggested that the arginine to leucine substitution disrupts all of these interactions (online supplemental figure 2E–G).

10.1136/jmedgenet-2020-107572.supp5 Supplementary data

**Figure 2:**
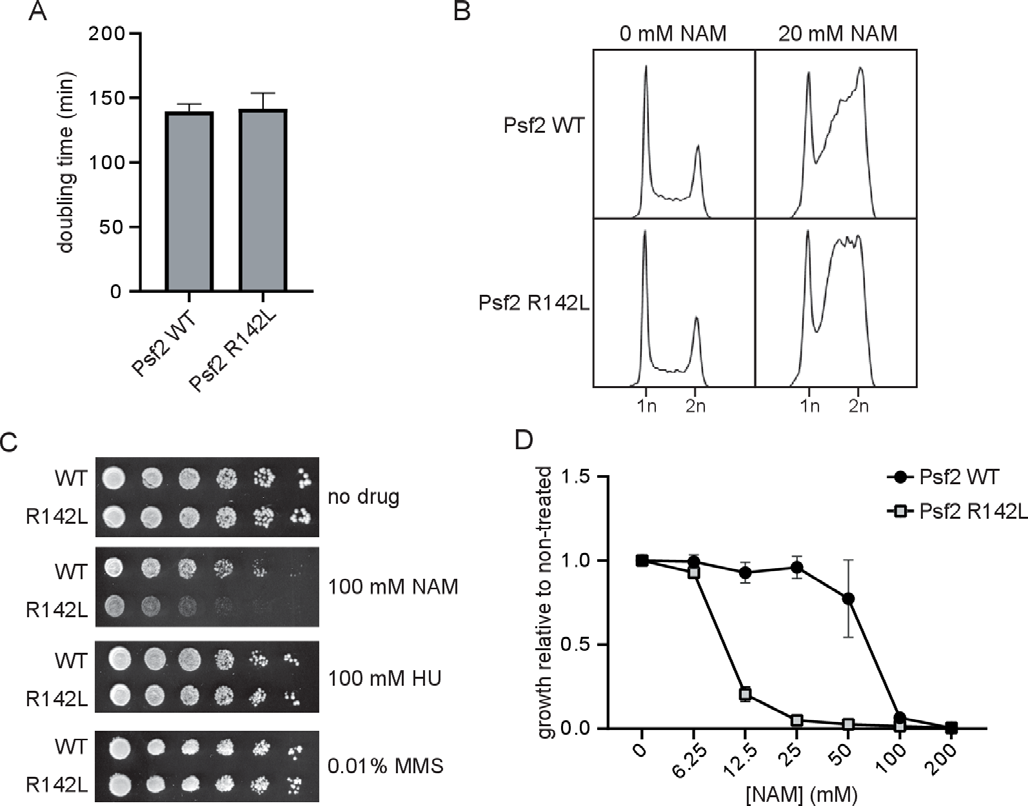
Strains of yeast expressing Psf2-R142L show reduced growth and altered cell cycle progression in the presence of nicotinamide (NAM). (A) OD_630_ of yeast cultures was monitored for 48 hours and doubling time was derived from exponential regression of the resulting growth curve (n=3). (B) Cell cycle profiles of actively replicating yeast cultures were assessed by flow cytometry after 8 hours of growth in the presence or absence of 20 mM NAM. (C) Serial fivefold dilutions of yeast were grown on solid media in the presence or absence of 100 mM NAM, 100 mM hydroxyurea (HU) or 0.01% methyl methanesulfonate (MMS) at 30°C for 72 hours (n=3). (D) Yeasts were cultured for 48 hours in the presence of a range of concentrations of NAM. Growth in the presence of NAM is presented as a fraction of growth in the absence of NAM (n=3). WT, wild type.

To evaluate the functional impact of the GINS2 p.(Arg114Leu) substitution, *S. cerevisiae* strains expressing Psf2 p.(Arg142Leu) (Psf2-R142L) were generated. No obvious differences in cell doubling time or cell cycle distribution were observed between strains expressing wild-type Psf2 (Psf2-WT) and those expressing Psf2-R142L on unperturbed growth (figure 2A, B). However, testing the effects of a series of compounds that induce DNA replication stress revealed that nicotinamide (NAM), a compound that causes DNA damage through inhibition of histone deacetylases of the sirtuin family, impaired the growth of Psf2-R142L-expressing cells (figure 2C, D). NAM-induced inhibition of the sirtuins Hst3 and Hst4 causes DNA damage in yeast. Moreover, *hst3∆ hst4∆* double mutation causes synthetic lethality when combined with epitope-tagged versions of DNA replication factors, indicating that subtle defects in DNA replication protein function can be detected using elevated NAM sensitivity as a read-out. While exposure to NAM resulted in the accumulation of cells in late S and G2 phases of the cell cycle for both Psf2-WT and Psf2-R142L-expressing cells (figure 2B), Psf2-R142L-expressing cells accumulated earlier in S phase than those expressing Psf2-WT, indicative of impaired DNA replication. Together, these observations indicate that the GINS2 p.(Arg113Leu) substitution negatively impacts the function of the corresponding protein.

So far, no other patient with *GINS2*-related MGORS has been identified, specifically using GeneMatcher or by contacting experts in this syndrome (see the Acknowledgements section for further details), corroborating the rarity of MGORS caused by pathogenic *GINS2* variants.

## Discussion

We describe a patient with growth delay, craniofacial dysmorphisms and craniosynostosis, and in whom a homozygous missense variant in the *GINS2* gene was identified. Although it is classified as of unknown clinical significance using the guidelines proposed by the American College of Medical Genetics and Genomics (ACMG), several lines of evidence support that this variant in *GINS2* is an additional cause of MGORS.

First, the homozygous p.(Arg114Leu) variant in *GINS2* is likely deleterious. This variant was identified in both healthy parents in heterozygosity. Since they are not consanguineous, they may share a very distant common ancestor, which is consistent with the homozygosity data. The high degree of intolerance of *GINS2* to homozygous variants (even of missense type) is suggestive of high selective pressure, thus supporting the possibility of *GINS2* being a disease-causing gene. Additionally, the *in silico* analysis also supported the pathogenicity of the p.(Arg114Leu) substitution, which occurred in a highly conserved residue in GINS2. Finally, modelling of the p.(Arg114Leu) substitution in the budding yeast *S. cerevisiae* showed that in yeast this substitution does not affect growth either under normal conditions or in the presence of hydroxyurea or methyl methanesulfonate, but confers sensitivity to DNA replication stress caused by the histone deacetylase inhibitor NAM, consistent with partially defective functions of Psf2 (yeast GINS2).

Second, this novel *GINS2* variant was identified in an individual with clinical features reminiscent of MGORS (online supplemental tables 3 and 4). She has the cardinal features of this syndrome, such as prenatal and postnatal growth restriction, patellar hypoplasia, microtia and coronal craniosynostosis. Additionally, she has overlapping skeletal, cardiac, gastrointestinal and anal abnormalities, and normal intelligence.

10.1136/jmedgenet-2020-107572.supp6 Supplementary data

10.1136/jmedgenet-2020-107572.supp7 Supplementary data

Third, the functional interactions of GINS2 and its role in DNA replication strengthen its causality in MGORS. GINS2 (OMIM*610609; GINS complex subunit 2) is part of the tetrameric GINS complex—composed of GINS1/GINS2/GINS3/GINS4—which is conserved in eukaryotes, from *S. cerevisiae* to *Homo sapiens*.^11–13^ This complex was shown to play an essential role in the initiation of DNA replication and progression of DNA replication forks,^11 12^ unwinding DNA for polymerase epsilon and binding preferentially to single-stranded DNA in the replicative helicase complex.^14^ The GINS tetramer interacts with CDC45 and MCM proteins to form the CMG helicase. The p.(Arg114Leu) substitution is located at the docking site of MCM5 and CDC45 at GINS2, which involves its N-terminal B-domain and its helical domain.^15^ The analysis of protein structural data suggested that this novel variant might compromise the interaction between GINS2 and both CDC45 and MCM5. Considering these subtle changes, we anticipated that the p.(Arg114Leu) missense variant might be hypomorphic, thus maintaining the partial function of GINS2. This is supported by the analysis of the equivalent Psf2-R142L substitution in yeast, which exhibited normal growth in several experimental conditions but a specific defect when exposed to NAM (figure 2).

Indeed, deletion of Psf1 in mice (*GINS1* in humans) results in early embryonic lethality.^12^ Of note, Psf1 is largely expressed in active stem cell systems in mice, including adult bone marrow, thymus, testis and ovary, but not the remaining adult tissues.^12^ Interestingly, five patients with compound heterozygous variants in *GINS1* were reported with neutropenia, natural killer cell deficiency and growth delay.^16^ Missense variants and variants located in the 5′ untranslated region resulted in lower GINS1 levels in patients’ cells, which showed impaired GINS complex assembly, basal replication stress, impaired checkpoint signalling, defective cell cycle control and genomic instability, which could be rescued by wild-type GINS1.^16^ Although our patient did not present immunodeficiency, growth retardation was evident, in particular in utero and during early infancy.

Our patient’s phenotype is strikingly similar to the phenotype of individuals with *CDC45* variants, particularly those who presented with craniosynostosis and mild MGORS features. Interestingly, pathogenic homozygous or compound heterozygous variants in the *CDC45* gene result in the distinctive MGORS clinical triad and frequently in craniosynostosis (OMIM#617063; MGORS7).^5^ Noteworthy, our patient had both patellae, though hypoplastic, and short stature was mild. Additionally, she developed cardiac and anal abnormalities. Also, pathogenic variants in *MCM5* and *MCM4*, other genes of the CMG helicase complex, were respectively associated with MGORS^6^ and a distinct growth delay phenotype.^17–20^ Based on the functional interaction between the CMG complex and DNA polymerases, we propose that changes in this complex would affect DNA replication as a possible pathophysiological mechanism for *GINS2*-related MGORS. In line with this assumption, biallelic hypomorphic missense variants in *GINS3* have been reported just recently, as an additional molecular cause of MGORS, suggesting defects in *GINS* genes as a cause of this clinical entity (Kannu *et al*, personal communication, 2020).

In summary, we report an individual with a homozygous likely disease-causing variant in *GINS2* and with clinical features overlapping those of MGORS, including prenatal and postnatal growth delay, hypoplastic patellae and typical craniofacial dysmorphisms, such as microtia and craniosynostosis. The recognition of the *GINS2* gene as a novel causative gene of MGORS is crucial for the anticipatory multidisciplinary care of affected individuals, as well as for genetic counselling, enabling parents the possibility of prenatal or preimplantation diagnosis. The apparent rarity of *GINS2* variants associated with MGORS may be explained because the phenotype arises only in a narrow window of disturbed GINS2 function, intermediate between lethal and normal outcomes.
