# Supplementary material for: Biallelic GINS2 variant p.(Arg114Leu) causes Meier-Gorlin syndrome with craniosynostosis
Source: J Med Genet. 2021 Aug 5;59(8):776–80. doi: 10.1136/jmedgenet-2020-107572 (PMC9340002; doi:10.1136/jmedgenet-2020-107572)
Supplement: Supplementary data [file jmedgenet-2020-107572supp001.pdf]

SUPPLEMENTARY METHODS

Exome sequencing analysis

Using DNA extracted from peripheral blood cells of the proband and both parents, exome capture of DNA was carried out using the SureSelect Human All Exon Kit v6 (Agilent) following the manufacturer's instructions. We generated a library for each sample using 3 μg DNA extracted from whole blood. Exome sequencing was performed on an Illumina HiSeq 4000, with 150 bp paired-end reads. The reads were mapped to the GRCh38 reference genome and artefacts excluded, as previously described.[1] We analysed the data assuming complete penetrance, allowing for the possibility of either a *de novo* mutation (dominant) or biallelic inheritance (recessive). We used a custom Platypus 0.8.1[2] Bayesian script to identify *de novo* mutations, and a script using bcftools 1.5 with the 1000G genetic map for calling regions of homozygosity. To identify biallelic variants we filtered on a minor allele frequency of <0.001 (The Genome Aggregation Database, gnomAD).[3] All variants called were checked by examining individual reads in GBrowse[4] and nonsynonymous variants assigned priority based on estimates of deleteriousness using Deleterious scores[5] and CADD scores.[6] Literature review of biochemistry data and the protein's functional association network was carried out to support a possible pathophysiological mechanism of a novel gene linked to MGORS.

Prediction of mutation impact on protein structure

To conduct *in silico* protein analysis the human (protein data bank, PDB ID: 6XTX[7]) and Saccharomyces cerevisiae (PDB IDs: 6SKL[8] and 3JC5[9]) CMG structures were retreived and visualized using both: i) Mol* (doi:10.2312/molva.20181103) tool imbedded in RCSB PDB, and ii) Maestro software (version 12.2.012, MMshare Version 4.8.012, Release 2019-4, Platform Darwin-x86_64). Structural prediction of wild-type GINS2/PSF2 and mutated

sequences was done using Maestro, HHPRED and MODeller softwares[10] for protein homology detection and structure prediction by comparative modeling of protein three-dimensional structures.

Yeast strains and growth conditions

Yeast strains used in this study are listed in Supplementary Table 5. Yeast strains were constructed and propagated using standard genetic methods. Yeast were cultured in YPD supplemented with adenine and incubated at 30˚C, unless otherwise indicated.

Generation of yeast strains

The *PSF2* gene, along with 500 bp of upstream and downstream sequence, was amplified from the genome of yeast BY4743 using primers p416-PSF2_Fwd and p416-PSF2_Rev and cloned into the plasmid p416GDP by gap repair to generate the plasmid p416-PSF2-WT (for primer sequences, see Supplementary Table 6). Site-directed mutagenesis of p416-PSF2-WT was performed using primers PSF2-R142L_Fwd and PSF2-R142L_Rev to generate plasmid p416-psf2-R142L. To generate cassettes of the 3' end of the *PSF2* gene linked to the *kanMX6* cassette for transformation of yeast, the 3' ends of *PSF2* from p416-PSF2-WT and p416-psf2-R142L were amplified using primers PSF2-C and PSF2-D, and the *kanMX6* cassette was amplified from pFA6a-kanMX6 [11] using primers PSF2-F2-STOP and PSF2-R1.[12] The PCR products of both reactions were then pooled and amplified with primers PSF2-C and PSF2-R1 to generate the cassettes 3'-*PSF2-WT::kanMX6* and *3'-psf2-R142L::kanMX6*, both of which encode the 3' end of the *PSF2* gene linked to the *kanMX6* cassette. Cassettes were used to transform yeast strain BY4741 to G418 resistance, and *PSF2* genes of isolates were sequenced by Sanger sequencing to confirm absence of undesired mutations.

Measurement of DNA content by flow cytometry

Cells were cultured for 8 hours at a density of less than 0.2 OD_{630} in the presence or absence of 20 mM NAM. Cells were fixed in 70% ethanol. Prior to analysis by flow cytometry, fixed cells were sonicated for 10 seconds at 30% duty cycle (Branson Digital Sonifier 450) and treated with 0.4 µg/mL RNAse A in 50 mM Tris-HCl pH 7.5 at 42˚C for 3 hours, followed by 1 mg/mL Proteinase K in 50 mM Tris-HCl pH 7.5 at 50˚C for 30 minutes. Cells were stained with Sytox Green (Invitrogen) to assess DNA content as previously described.[13] Analysis was performed using a FACS Calibur flow cytometer with CellQuest Pro software (BD Biosciences). Further analyses were performed using FlowJo software (version 10.6.2, BD Biosciences).

Assay for cell doubling time

Yeast cultures were diluted to OD_{630} 0.001 and 100 µL aliquots were transferred to a 96-well plate. Cultures were incubated at 30˚C for 48 hours in a BioTek ELx800 plate reader with Gen5 software (ver 2.09, BioTek Instruments). Culture density was monitored at 30 minute intervals by measuring the OD_{630} after 30 seconds of agitation to ensure that cells were homogenously mixed. Doubling times were derived from exponential regression of the resulting growth curve.

Drug susceptibility assay

Yeast cultures were diluted to the same OD_{630}, and 100 µL aliquots placed in a 96-well plate. A series of 5-fold dilutions was prepared, and spotted onto the indicated solid media using a replica plater tool (Sigma-Aldrich). Growth of yeast was recorded every 24 hours for 4 days.

Assay for minimum inhibitory concentration of NAM

Yeast cultures were diluted to 0.0005 OD_{630} and 100 µL aliquots were incubated in 96-well with a range of concentrations of nicotinamide (NAM). NAM is a compound that causes DNA damage through inhibition of histone deacetylases of the sirtuin family.[14] NAM-induced inhibition of the sirtuins Hst3 and Hst4 causes DNA damage in yeast.[14,15] Moreover, *hst3*Δ *hst4*Δ double mutation causes synthetic lethality when combined with epitope-tagged versions of DNA replication factors,[16,17] indicating that subtle defects in DNA replication protein function can be detected using elevated NAM sensitivity as a readout. OD_{630} was measured after 48 hours using an EL800 plate reader with Gen5 software (ver 2.09, BioTek Instruments). Growth of yeast was normalized to an untreated control well for each strain.

References:

1. Miller KA, Twigg SR, McGowan SJ, Phipps JM, Fenwick AL, Johnson D, Wall SA, P Noons, Rees KEM, Tidey EA, Craft J, Taylor J, Taylor JC, Goos JAC, Swagemakers SMA, Mathijssen IMJ, van der Spek PJ, Lord H, Lester T, Abid N, Cilliers D, Hurst JA, Morton JEV, Sweeney E, Weber A, Wilson LC, Wilkie AOM. Diagnostic value of exome and whole genome sequencing in craniosynostosis. *J Med Genet* 2017;54:260-68.

2. Rimmer A, Phan H, Mathieson I, Iqbal Z, Twigg SRF; WGS500 Consortium, Wilkie AOM, McVean G, Lunter G. Integrating mapping-, assembly- and haplotype-based approaches for calling variants in clinical sequencing applications. *Nat Genet* 2014;46:912-918.

3. Karczewski KJ, Francioli LC, Tiao G, Cummings BB, Alfoldi J, Wang Q, Collins RL, Laricchia KM, Ganna A, Birnbaum DP, Gauthier LD, Brand H, Solomonson M, Watts NA, Rhodes D, Singer-Berk M, England EM, Seaby EG, Kosmicki JA, Walters RK, Tashman K, Farjoun Y, Banks E, Poterba T, Wang A, Seed C, Whiffin N, Chong JX, Samocha KE, Pierce-Hoffman E, Zappala Z, O'Donnell-Luria AH, Minikel EV, Weisburd B, Lek M, Ware JS, Vittal C, Armean IM, Bergelson L, Cibulskis K, Connolly KM, Covarrubias M, Donnelly S, Ferriera S, Gabriel S, Gentry J, Gupta N, Jeandet T, Kaplan D, Llanwarne C, Munshi R, Novod S, Petrillo N, Roazen D, Ruano-Rubio V, Saltzman A, Schleicher M, Soto J, Tibbetts K, Tolonen C, Wade G, Talkowski ME, Genome Aggregation Database Consortium; Neale BM, Daly MJ, MacArthur DG. The mutational constraint spectrum quantified from variation in 141,456 humans. *Nature* 2020;581:434-443.

4. Stein LD, Mungall C, Shu S, Caudy M, Mangone M, Day A, Nickerson E, Stajich JE, Harris TW, Arva A, Lewis S. The generic genome browser: a building block for a model organism system database. *Genome Res* 2002;12:1599-610.

5. Calpena E, Cuellar A, Bala K, Swagemakers SMA, Koelling N, McGowan SJ, Phipps JM, Balasubramanian M, Cunningham ML, Douzgou S, Lattanzi W, Morton JEV, Shears D, Weber A, Wilson LC, Lord H, Lester T, Johnson D, Wall SA, Twigg SRF, Mathijssen IMJ, Boardman-Pretty F; Genomics England Research Consortium, Boyadjiev SA, Wilkie AOM. *SMAD6* variants in craniosynostosis: genotype and phenotype evaluation. *Genet Med* 2020;22:1498-506.

6. Rentzsch P, Witten D, Cooper GM, Shendure J, Kircher M. CADD: predicting the deleteriousness of variants throughout the human genome. *Nucleic Acids Res* 2019;47:D886-D894.

7. Rzechorzek NJ, Hardwick SW, Jatikusumo VA, Chirgadze DY, Pellegrini L. CryoEM structures of human CMG-ATPgammaS-DNA and CMG-AND-1 complexes. *Nucleic Acids Res* 2020;48:6980-6995.

8. Baretic D, Jenkyn-Bedford M, Aria V, Cannone G, Skehel M, Yeeles JTP. Cryo-EM Structure of the Fork Protection Complex Bound to CMG at a Replication Fork. *Mol Cell* 2020;78:926-940.e13.

9. Yuan Z, Bai L, Sun J, Georgescu R, Liu J, O'Donnell ME, Li H. Structure of the eukaryotic replicative CMG helicase suggests a pumpjack motion for translocation. *Nat Struct Mol Biol* 2016;23:217-24.

10. Zimmermann L, Stephens A, Nam SZ, Rau D, Kubler J, Lozajic M, Gabler F, Soding J, Lupas AN, Alva V. A Completely Reimplemented MPI Bioinformatics Toolkit with a New HHpred Server at its Core. *J Mol Biol* 2018;430:2237-2243.

11. Bähler J, Wu J-Q, Longtine MS, Shah NG, McKenzie A, Steever AB, Wach A, Philippsen P, Pringle JR. Heterologous modules for efficient and versatile PCR-based gene targeting in *Schizosaccharomyces pombe*. *Yeast* 1998;14:943-951.

12. Longtine MS, McKenzie A, Demarini DJ, Shah NG, Wach A, Brachat A, Philippsen P, Pringle JR. Additional modules for versatile and economical PCR-based gene deletion and modification in *Saccharomyces cerevisiae*. *Yeast* 1998;14:953-961.

13. Haase SB, Reed SI. Improved flow cytometric analysis of the budding yeast cell cycle. *Cell Cycle* 2002;1:117-121.

14. Simoneau A, Ricard É, Weber S, Hammond-Martel I, Wong LH, Sellam A, Giaever G, Nislow C, Raymond M, Wurtele H. Chromosome-wide histone deacetylation by sirtuins prevents hyperactivation of DNA damage-induced signaling upon replicative stress. *Nucleic Acids Res.* 2016;44:2706-26.

15. Celic I, Masumoto H, Griffith WP, Meluh P, Cotter RJ, Boeke JD, Verreault A. The sirtuins hst3 and Hst4p preserve genome integrity by controlling histone h3 lysine 56 deacetylation. *Curr Biol.* 2006;16:1280-9.

16. Celic I, Verreault A, Boeke JD. Histone H3 K56 hyperacetylation perturbs replisomes and causes DNA damage. *Genetics.* 2008;179:1769-84.

17. Simoneau A, Delgoshaie N, Celic I, Dai J, Abshiru N, Costantino S, Thibault P, Boeke JD, Verreault A, Wurtele H. Interplay between histone H3 lysine 56 deacetylation and chromatin modifiers in response to DNA damage. *Genetics.* 2015;200:185-205.
