# Supplementary material for: Biallelic GINS2 variant p.(Arg114Leu) causes Meier-Gorlin syndrome with craniosynostosis
Source: J Med Genet. 2021 Aug 5;59(8):776–80. doi: 10.1136/jmedgenet-2020-107572 (PMC9340002; doi:10.1136/jmedgenet-2020-107572)
Supplement: Supplementary data [file jmedgenet-2020-107572supp008.pdf]

Supplementary Table 5. Genotypes of yeast strains used in this study.

Strain	Genotype	Reference
BY4743	BY4743 *MAT a/alpha his3Δ1/his3Δ1 leu2Δ0/leu2Δ0 LYS2/lys2Δ0 met15Δ0/MET15 ura3Δ0/ura3Δ0*	PMID: 9483801.
BY4741	BY4741 *MAT a his3Δ1/his3Δ1 leu2Δ0/leu2Δ0 ura3Δ0/ura3Δ0*	PMID: 9483801.
MMY5811	BY4741 *PSF2-WT::kanMX*	This study
MMY5815	BY4741 *psf2-R142L::kanMX*	This study

Reference:

Brachmann CB, Davies A, Cost GJ, Caputo E, Li J, Hieter P, Boeke JD. Designer deletion strains derived from *Saccharomyces cerevisiae* S288C: A useful set of strains and plasmids for PCR-mediated gene disruption and other applications. *Yeast* 1998;14:115-132. PMID: 9483801.
