# Supplementary material for: Biallelic GINS2 variant p.(Arg114Leu) causes Meier-Gorlin syndrome with craniosynostosis
Source: J Med Genet. 2021 Aug 5;59(8):776–80. doi: 10.1136/jmedgenet-2020-107572 (PMC9340002; doi:10.1136/jmedgenet-2020-107572)
Supplement: Supplementary data [file jmedgenet-2020-107572supp009.pdf]

BMJ Publishing Group Limited (BMJ) disclaims all liability and responsibility arising from any reliance placed on this supplemental material which has been supplied by the author(s)

Supplementary Table 6. Oligonucleotide primers used in this study.

Primer	Sequence
p416-PSF2_Fwd	CTCACTAAAGGGAACAAAAGCTGGAGCTCAGTTTATCTCTTTATGACACTTGAAAAC
p416-PSF2_Rev	CAAGGCGATTAAGTTGGGTAACGCCAGGGTTTTCCCAACAACAACAGCAGCAACAAC
PSF2-R142L_Fwd	CCCATTCACGAATTACTTGGTAAAATACAAGAC
PSF2-R142L_Rev	GTCTTGTATTTTACCAAGTAATTCGTGAATGGG
PSF2-C	TTTAAATAATATGGTAGCCATGCGT
PSF2-D	ACCATAACGACAAACAAGAATCAAT
PSF2-F2-STOP	CACAGCATCTCTTACCGCTGGTACTGAAAATGATGAAGAAGAATTCAATATTTAACGGATCCCCGGGTTAATTAA
PSF2-R1	ATATATACATACGCATATCACTGAAAGTTCTAATTATTGCAAAGCCAGCACTTTTGAATTCGAGCTCGTTTAAAC
