# Supplementary material for: Biallelic GINS2 variant p.(Arg114Leu) causes Meier-Gorlin syndrome with craniosynostosis
Source: J Med Genet. 2021 Aug 5;59(8):776–80. doi: 10.1136/jmedgenet-2020-107572 (PMC9340002; doi:10.1136/jmedgenet-2020-107572)
Supplement: Supplementary data [file jmedgenet-2020-107572supp002.pdf]

Supplementary table 1. Length/height, weight and head circumference of girl with a homozygous missense *GINS2* variant.

Age	Lenght/height (cm)	Pct	SD	Weight (kg)	Pct	SD	Head circumference (cm)	Pct	SD
Birth	47	14	-1.10	2.260	1	-2.22	30.5	< 1	-2.92
3 months	57	18	-0.90	4.270	3	-1.85	36	< 1	-3.08
6 months	62	12	-1.19	5.210	1	-2.51	39	< 1	-2.76
9 months	65	5	-1.69	5.760	< 1	-3.15	42	6	-1.55
12 months	68	3	-1.89	6.640	< 1	-3.10	43	6	-1.60
18 months	73.5	2	-2.02	7.510	< 1	-3.42	45.3	19	-0.88
22 months	79	8	-1.39	8.400	< 1	-3.08	45.5	12	-1.19
2y 9m	87.2	7	-1.45	10,500	1	-2.24	46.5	12	-1.19
3y 2m	89.5	7	-1.50	11.400	3	-1.96	47.6	24	-0.72
3y 6m	92.5	11	-1.21	11.000	1	-2.53	NA	na	na
4y 1m	96.7	16	-1.00	12.400	2	-2.10	NA	na	na
5y	101.7	11	-1.20	12.950	1	-2.51	NA	na	na
5y 7m	105.7	12	-1.16	14.200	1	-2.31	NA	na	na
6y	111	26	-0.64	15.100	1	-2.19	NA	na	na
7y	117.5	26	-0.64	17.300	3	-1.95	NA	na	na

Pct, Percentile. SD, Standard deviation. y, years. m, months. NA, not available. na, not applicable.
Reference: https://simulconsult.com/resources/measurement.html
