# Supplementary material for: Biallelic GINS2 variant p.(Arg114Leu) causes Meier-Gorlin syndrome with craniosynostosis
Source: J Med Genet. 2021 Aug 5;59(8):776–80. doi: 10.1136/jmedgenet-2020-107572 (PMC9340002; doi:10.1136/jmedgenet-2020-107572)
Supplement: Supplementary data [file jmedgenet-2020-107572supp004.pdf]

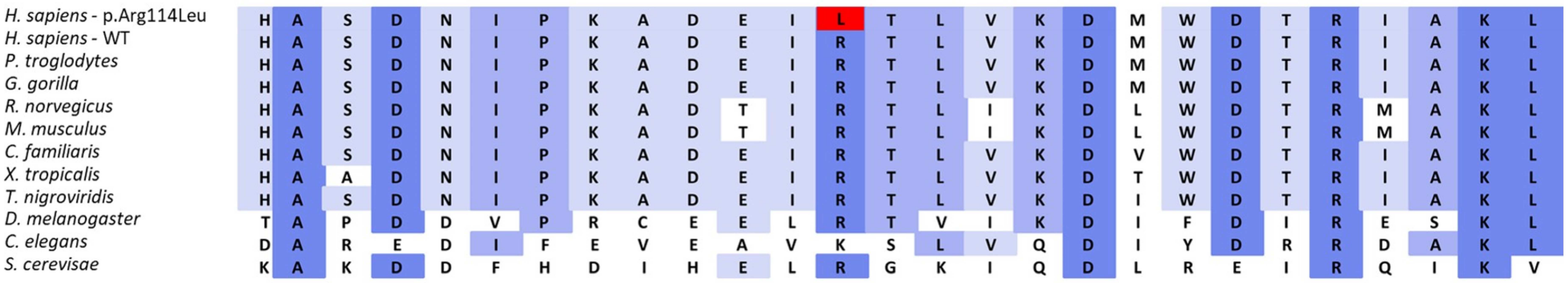
H. sapiens - p.Arg114Leu H A S D N I P K A D E I L T L V K D M W D T R I A K L
H. sapiens - WT H A S D N I P K A D E I R T L V K D M W D T R I A K L
P. troglodytes H A S D N I P K A D E I R T L V K D M W D T R I A K L
G. gorilla H A S D N I P K A D E I R T L V K D M W D T R I A K L
R. norvegicus H A S D N I P K A D T I R T L I K D L W D T R M A K L
M. musculus H A S D N I P K A D T I R T L I K D L W D T R M A K L
C. familiaris H A S D N I P K A D E I R T L V K D V W D T R I A K L
X. tropicalis H A A D N I P K A D E I R T L V K D T W D T R I A K L
T. nigroviridis H A S D N I P K A D E I R T L V K D I W D T R I A K L
D. melanogaster T A P D D V P R C E E L R T V I K D I F D I R E S K L
C. elegans D A R E D I F E V E A V K S L V Q D I Y D R R D A K L
S. cerevisae K A K D D F H D I H E L R G K I Q D L R E I R Q I K V
