# Supplementary material for: Biallelic GINS2 variant p.(Arg114Leu) causes Meier-Gorlin syndrome with craniosynostosis
Source: J Med Genet. 2021 Aug 5;59(8):776–80. doi: 10.1136/jmedgenet-2020-107572 (PMC9340002; doi:10.1136/jmedgenet-2020-107572)

Supplementary figure 2A

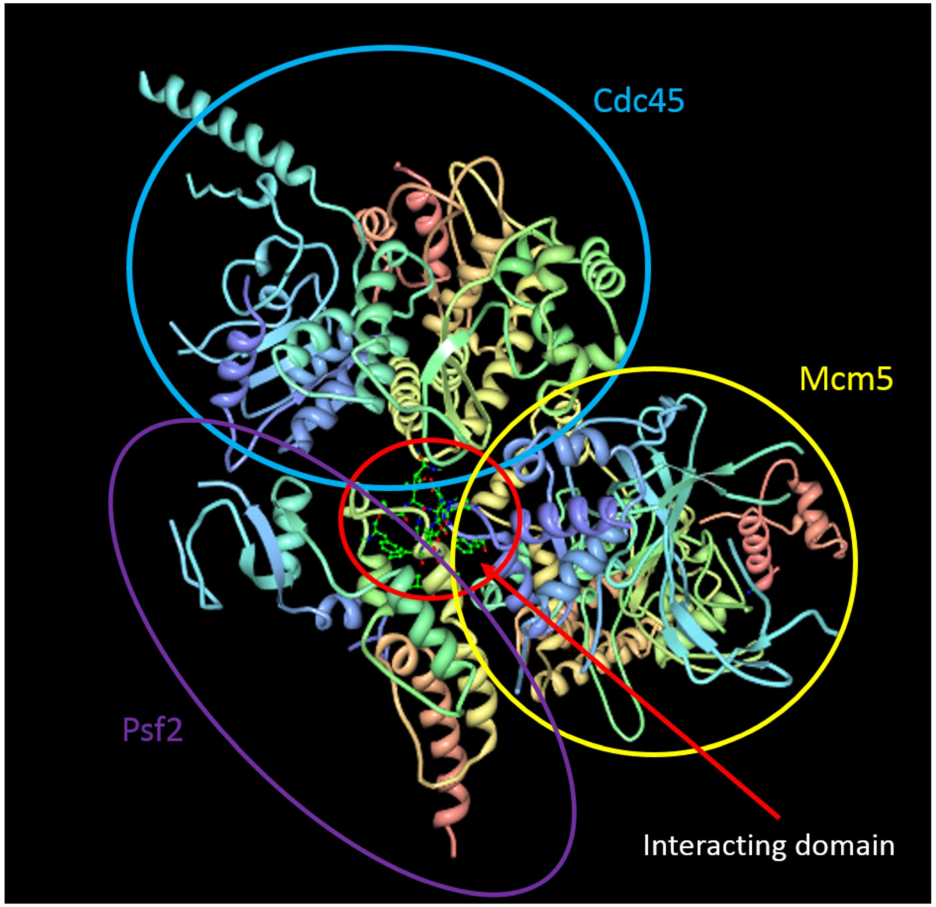

Mcm5 interactions:

NP_006730.2	44	VGTDRTGFTFKYRDELKRHYNLGEYWIEVEMEDLASFDEDLADYLYKQPA	93
XP_001156025.1	44	VGTDRTGFTFKYRDELKRHYNLGEYWIEVEMEDLASFDEDLADYLYKQPA	93
XP_005626282.1	1	------MVMMWAQDELKRHYNLGEYWVEVEMEDLASFDEDLADYLYKQPA	44
NP_001068758.1	44	VGTDRTGFTFKYRDELKRHYNLGEYWIEVEMEDLASFDEELADYLYKQPA	93
NP_032592.2	44	VGTDRTGFTFKYRDELKRHYNLGEYWIEVEMEDLASFDEELADHLHKQPA	93
NP_001099640.1		--	
NP_001006243.1	44	VGTDRTGFTFKYRDELKRHYNLGQYWVEVEMEDLASFDEDLADYLYKQPT	93
NP_848523.2	45	VGTDRTGFTYKYRDELKRHYTLGEYWIEVEMEDLASFDEDLSDCLYKLPS	94
NP_524308.2	42	---NEENFFYKYRDTLKRNYLNGRYFLEIEMEDLVGFDETLADKLNKQPT	88
XP_313694.2	43	---CEANFSYKYRDTLKRNYLLGRYYLEVEIEDLAGFDESLADKLYKQPT	89
NP_497858.1	47	---STGGFGMIYRDQLKRNYFSHEYRLEINLNHLKNFDEDIEMKLRKFPG	93
NP_013376.1	39	----RLDSQFIYRDQLRNNILVKNYSLTVNMEHLIGYNEDIYKKLSDEPS	84
XP_453475.1	39	----RLDSNFVYREQLRNNLLVHKYFLDVNTEHLIGYNEDLYKKLQDEPI	84
NP_986942.1	39	----RLDARFVYREQLRNNLLVRRYALRVNTEHLIGYNEALYKLVRDEPV	84
XP_001713071.1	42	----VIDNDFIYRTQLRDNLVVKQYMLNIDLRHLISYNEDLAHLLLSQPT	87
XP_003711077.1	38	----RLENKFIYRDQLRENALLGAYYCDVNIGDLIKFNEELAHRIVTEPT	83
XP_961537.1	41	----RLDNSFIYRDQLRENALLKKYYCDVNIGDLIKFNEEIAHRLVTEPA	86
NP_178812.1	43	---EIEQNCFPYREALLDN----PKRLVVHLEDLLSFDSDLPSLIRSAPA	85
NP_001048396.1	47	---TGPTGDFPYRESLVHN----RDHVTVAIEDLDAFDAELSDKIRKSPA	89
NP_001017327.2	45	VGTDRTGFTFKYRDELKRHYNLGEYWIEVEMEDLASFDEDLADYLYKQPT	94

Cdc45 interactions:

NP_001171481.1	297	VDCTRISFEYDLRLVLYQHWSLHDSLCNTSYTAARFKLWSVHGQKRLQEF	346
XP_003317140.1	297	VDCTRISFEYDLRLVLYQHWSLHDSLCNTSYTAARFKLWSVHGQKRLQEF	346
XP_001104872.2	297	VDCTRISFEYDLRLALYQHWSLHDSLCNTSYTAARFKLWSVHGQKRLQEF	346
XP_543547.3	268	VDCTRISFEYDLRLALYQHWSLHDSLCNTCYTAARFKLWSVHGQKRLQEF	317
NP_001019661.1	266	VDCARISFEYDLRLALYQHWSLHDSLCNTCYTAARLQLWSLHGQKRLQEF	315
NP_033992.2	265	VDCTRISFEYDLCLVLYQHWSLHESLYNTSYTAARFKLWSVHGQKRLQEF	314
NP_001099336.1	269	VDCTRISFEYDLCLVLYQHWSLHESLYNTSYTAARFKLWSVHGQKRLQEF	318
XP_415070.2	265	IDCMRIAFEYDLRLALYQHWSLYESLCNTSYTSATLKLWSVQGQKRLQEF	314
NP_998551.1	271	IDCMRINFEYDLRLVLYQHWSLYESICNSCYTSCSFKLWSINGQKKLQEF	320
NP_569880.1	278	MSASKITFENDLHLVLYRHWPVTESMRYSRYSSCQLKLWTLRGEKRLHEL	327
XP_320573.1	278	QTSVKIVFESDLQLALFRHWSVLDSLRYSYYPACRLKLWTHKGDKQMNEL	327
NP_497756.2	267	DDLLHITFGRELPLALYSHWDLFSAMMVSEYFSIKTKNWTQKGDVNIRHL	316
NP_013204.1	313	PDTLTLNIQPDYYLFLLRHSSLYDSFYYSNYVNAKLSLWNENGKKRLHKM	362
XP_455970.1	316	PDSLNLEIRPDYSLFLLRHTSLYDSFYYSNYVNAKLSLWNENGKKRLHKM	365
NP_986736.1	281	ADKLSIDVRPDYLLFLMRHSSLYDSFFYSNYVNARLSLWNENGKKRFHKM	330
NP_594693.1	302	PHDQSIRLEDEFRFMLVRHWSLYDSMLHSAYVGSRLHIWSEEGRKRLHKL	351
XP_003714523.1	449	PEDTSIRLSPEPKFLLIRHWSLYDSMLHSPYLFSRLKMWSETGLKRLHKL	498
XP_957455.1	429	PEDTSIRLSPEPKFLLIRHWSLYDSMLHSPYLFSRLKTWSEAGMKRLHKL	478
NP_189146.1	298	PDCSRISYEEEPRLMLLREWTLFDSMLCSSYIATKLKTWSDNGIKKLKLL	347
NP_001065635.1	302	PEASRIAYEDEPRLMLLREWSLFDSMLCSSYVATKLKTWSDNGLKKLKLL	351
NP_001066041.1	302	PEASRIAYEDEPRLMLLREWSLFDSMLCSSYVATKLKTWSDNGLKKLKLL	351
XP_004910630.1	268	IDCMRIAFEYDLRLSLYQHWSLYESICNSCYTSASLKLWSVQGQKKLQEF	317

Supplementary figure 2B

```
NP_057179.1      ----------MDAAEVEFLAEKELVTIIPNFSLDKIYLIGGD------------------ 32
NP_012463.1      MSLPAHLQQTFSPEEIQFIVENEPIKIFPRITTRQK-IRGDDRGTGNHTRWQLITTDDKA 59
                           :.  *::*:.*:* :.*:*.::  :  : *.*

NP_057179.1      LGPFNPGLPVEVPLWLAINLKQRQKCRLLPPEWMDVEKLEKMRDHER-KEETFTPMPSPY 91
NP_012463.1      LNNMVAMRSTEVVLWIALLLKQQSKCSIVAPQWLTTKELDRKIQYEKTHPDRFSELPWNW 119
                 *. :     .** **:*: ***:.** :: *:*: .::*::  ::*: : : *: :*  :

NP_057179.1      YMELTKLLLNHASDNIPK-ADEIRTLVKDMWDTRIAKLRVSADSFVRQQEAHAKLDNLTL 150
NP_012463.1      -LVLARILFNKAKDDFHDPIHELRGKIQDLREIRQIK---VLKGLKYLNESHLQLDNLSL 175
                  : *:::*:*:*.*:: .  .*:*  ::*: : *  *     ..:   :*:* :****:*

NP_057179.1      MEINTSGTFLTQALNHMYKLRTNLQPLES-TQSQDF--  185
NP_012463.1      LEINELRPFITEIMDKLREIHTASLTAGTENDEEEFNI  213
```

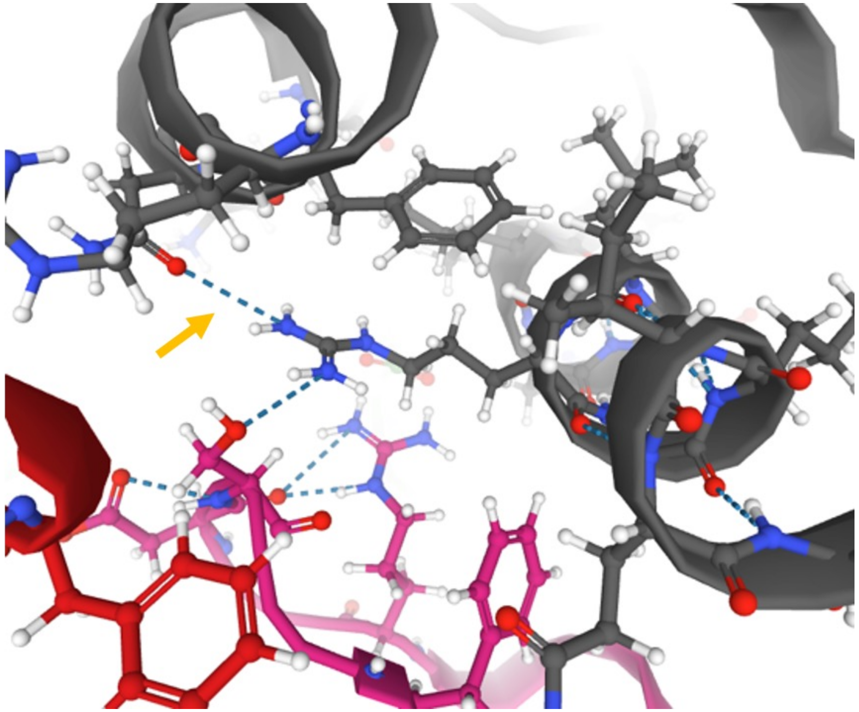

Supplementary figure 2C

```
Query  5    ISQFSEAYNKILRNSSSHSSCQLVIFVSCLNIDALCATKMLSLLFKKQLVQSQIVPIFGY  64
            +S F + + +++++       ++++FV+  ++DALCA K+L  LF+   VQ  +VP+ G+
Sbjct  3    VSDFRKEFYEVVQSQ------RVLLFVAS-DVDALCACKILQALFQCDHVQYTLVPVSGW  55

Query  65   SELRRHYSQLDDNINSLLLVGFGGVIDLEAFLEIDPQEYVIDTDEKSGEQSFRRDIYVLD  124
             EL   + +  +  +L+  G  +DL   L+  P E  I               +V D
Sbjct  56   QELETAFLEHKEQFHYFILINCGANVDLLDILQ--PDEDTI--------------FFVCD  99

Query  125  AHRPWNLDNIFGSQIIQCFDDGTVDDTLGEQKEAYYKLLELDEESGDDELSGDENDNNGG  184
             HRP N+ N++    I+       DD L  +  AY  +   +EE  D+E SG+++D +
Sbjct  100  THRPVNVVNVYNDTQIKLLIKQ--DDDL--EVPAYEDIFRDEEE--DEEHSGNDSDGSEP  153

Query  185  DDEATDADE--VTDEDEEDEDETISNKRGNSS--------------IGP--NDLSKRKQR  226
             ++ T  +E  V       +      + G+ S               GP  +  + R  R
Sbjct  154  SEKRTRLEEEIVEQTMRRRQRREWEARSGSGSEPVAAALEKSSRLFAGPMSDRTAPRSPR  213

Query  227  KKQIHEYEGVLEEYYSQGTTVVNSISAQIYSLLSAIGETNLSN-LWLNILGTTSL---DI  282
            +  + +YE    +Y   GT+     SA +   L+ +   +L++ LW  I+G T     D
Sbjct  214  RDILFDYE----QYEYHGTS-----SAMVMFELAWMLSKDLNDMLWWAIVGLTDQWVQDK  264

Query  283  AYAQVYNRLYPLLQDEVKRLTPSSRNSVKT--PDTLTLNIQPDYYLFLLRHSSLYDSFYY  340
                 Y     +LQ  V R    + +   T   D   ++ + D  L L +H SL+DS
Sbjct  265  ITQMKYVTDVGVLQRHVSRHNHRNEDEENTLSVDCTRISFEYDLRLVLYQHWSLHDSLCN  324

Query  341  SNYVNAKLSLWNENGKKRLHKMFARMGIPLSTAQETWLYMDHSIKRELGIIFDKNLDRYG  400
            ++Y  A+  LW+ +G+KRL +  A MG+PL   ++ +  MD S+K  L  + +++ +++G
Sbjct  325  TSYTAARFKLWSVHGQKRLQEFLADMGLPLKQVKQKFQAMDISLKENLREMIEESANKFG  384

Query  401  LQDIIRDGFVRTLGYRGSISASEFVEALTALLEVGNSTDKDSVKINNDNNDDTDGEEEED  460
            ++D+    F    G++    AS+ V A  +L+E   S +KD           TD
Sbjct  385  MKDMRVQTFSIHFGFKHKFLASDVVFATMSLME---SPEKD--------GSGTD------  427

Query  461  NSAQKLTNLRKRWVSNFWLSWDALDDRKVELLNRGIQLAQDLQRAIFNTGVAILEKKLIK  520
                           +F  + D+L    ++ L  G++LA+   RA   T  + L   L+
Sbjct  428  ---------------HFIQALDSLSRSNLDKLYHGLELAKKQLRATQQTIASCLCTNLVI  472

Query  521  HLRIYRLCVLQDG-PDLDLYRNPLTLLRLGNWLIE---CCAESEDKQLLPMVLAS-IDEN  575
                +  C L +G PD+ L+  P +L  L   L++   C  ++   +LLP+V+A+ +
Sbjct  473  SQGPFLYCSLMEGTPDVMLFSRPASLSLLSKHLLKSFVCSTKNRRCKLLPLVMAAPLSME  532

Query  576  TDTYLVAGLTPRYPRGLDTIHTKKPILNNFSMAFQQITAETDAKVRIDNFESSIIEIRRE  635
              T  V G+ P      D+   K    N F  AF++    T +++  ++F+ S+IE++ E
Sbjct  533  HGTVTVVGIPPE----TDSSDRK----NFFGRAFEKAAESTSSRMLHNHFDLSVIELKAE  584

Query  636  DLSPFLEKL  644
            D S FL+ L
Sbjct  585  DRSKFLDAL  593
```

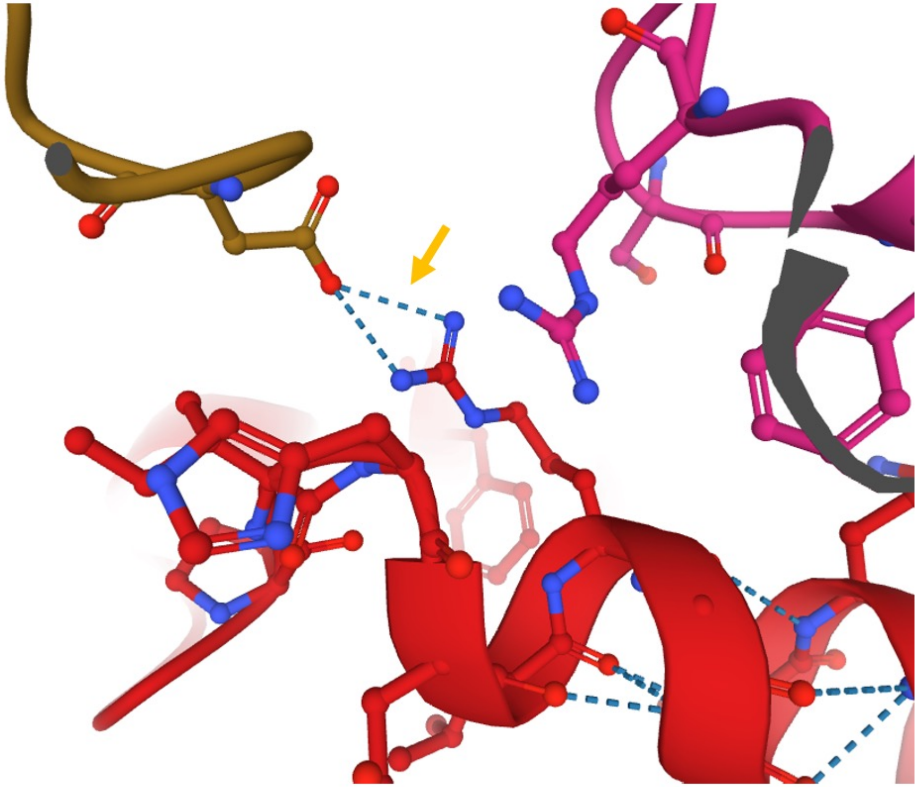

Supplementary figure 2D

```
Query  3    FDRPEIYSAPVLQGESPNDDDN---TEIIKSFKNFILEFRLDSQ-----FIYRDQLRNNI  54
            FD P I+ +    G++  D+     +++ + FK F+ ++R+ +      F YRD+L+ +
Sbjct  4    FDDPGIFYSDSFGGDAQADEGQARKSQLQRRFKEFLRQYRVGTDRTGFTFKYRDELKRHY  63

Query  55   LVKNYSLTVNMEHLIGYNEDIYKKLSDEPSDIIPLFETAITQVAKRISILSRAQSANNND  114
             +  Y + V ME L  ++ED+   L  +P++ + L E A  +VA ++
Sbjct  64   NLGEYWIEVEMEDLASFDEDLADYLYKQPAEHLQLLEEAAKEVADEVT------------  111

Query  115  KDPENTSMDTDSLLLNSLPTFQLILNSNANQIPLRDLDSEHVSKIVRLSGIIISTSVLSS  174
              P + +          L   Q++L S+A+   +R L S+ +S +V++ GIII+ S + +
Sbjct  112  -RPRPSGEEV-------LQDIQVMLKSDASPSSIRSLKSDMMSHLVKIPGIIIAASAVRA  163

Query  175  RATYLSIMCRNCRHTTSITINNFNSITG-NTVSLPRSCLSTIESESSMANESNIGDESTK  233
            +AT +SI CR+CR+T    + N     G    +LPR C +               D++ +
Sbjct  164  KATRISIQCRSCRNT----LTNIAMRPGLEGYALPRKCNT---------------DQAGR  204

Query  234  KNCGPDPYIIIHESSKFIDQQFLKLQEIPELVPVGEMPRNLTMTCDRYLTNKVIPGTRVT  293
              C  DPY I+ +  K +D Q LKLQE+P+ VP GEMPR++ + CDRYL +KV+PG RVT
Sbjct  205  PKCPLDPYFIMPDKCKCVDFQTLKLQELPDAVPHGEMPRHMQLYCDRYLCDKVVPGNRVT  264

Query  294  IVGIYSIYnskngagsgrsgggnggsgVAIRTPYIKILGIQSDVETSSIWNSVTMFTeee  353
            I+GIYSI           +  G    GV IR+ YI++LGIQ D + S   +     + +E
Sbjct  265  IMGIYSI-----KKFGLTTSRGRDRVGVGIRSSYIRVLGIQVDTDGSGR-SFAGAVSPQE  318

Query  354  eeeFLQLSRNPKLYEILTNSIAPSIFGNEDIKKAIVCLLMGGSKKILPDGMRLRGDINVL  413
            EEEF +L+  P +YE+++ SIAPSIFG  D+KKAI CLL GGS+K LPDG+  RGDIN+L
Sbjct  319  EEEFRRLAALPNVYEVISKSIAPSIFGGTDMKKAIACLLFGGSRKRLPDGLTRRGDINLL  378

Query  414  LLGDPGTAKSQLLKFVEKVSPIAVYTSGKGSSAAGLTASVQRDPMTREFYLEGGAMVLAD  473
            +LGDPGTAKSQLLKFVEK SPI VYTSGKGSSAAGLTASV RDP +R F +EGGAMVLAD
Sbjct  379  MLGDPGTAKSQLLKFVEKCSPIGVYTSGKGSSAAGLTASVMRDPSSRNFIMEGGAMVLAD  438

Query  474  GGVVCIDEFDKMRDEDRVAIHEAMEQQTISIAKAGITTVLNSRTSVLAAANPIYGRYDDL  533
            GGVVCIDEFDKMR++DRVAIHEAMEQQTISIAKAGITT LNSR SVLAAAN ++GR+D+
Sbjct  439  GGVVCIDEFDKMREDDRVAIHEAMEQQTISIAKAGITTTLNSRCSVLAAANSVFGRWDET  498

Query  534  KSPGDNIDFQTTILSRFDMIFIVKDDHNEERDISIANHVINIHTGnanamqnqqeenGSE  593
            K   DNIDF  TILSRFDMIFIVKD+HNEERD+ +A HVI +H       Q  +     E
Sbjct  499  KGE-DNIDFMPTILSRFDMIFIVKDEHNEERDVMLAKHVITLHVSALTQTQAVE----GE  553

Query  594  ISIEKMKRYITYCRLKCAPRLSPQAAEKLSSNFVTIRKQLLINELESTERSSIPITIRQL  653
            I + K+K++I YCR+KC PRLS +AAEKL + ++ +R     +E +S  RSSIPIT+RQL
Sbjct  554  IDLAKLKKFIAYCRVKCGPRLSAEAAEKLKNRYIIMRSGARQHERDSDRRSSIPITVRQL  613

Query  654  EAIIRITESLAKLELSPIAQERHVDEAIRLFQASTMDAASQDPIGGLNQASGTSLSE-IR  712
            EAI+RI E+L+K++L P A E  V+EA+RLFQ ST+DAA    + G+   +     E +
Sbjct  614  EAIVRIAEALSKMKLQPFATEADVEEALRLFQVSTLDAALSGTLSGVEGFTSQEDQEMLS  673

Query  713  RFEQELKRRLPIGWSTSYQTLRREFVDTHRFSQLALDKALYALEKHETIQLRHQGQNIYR  772
            R E++LKRR  IG   S  ++ ++F    ++ + A+ K L  + +   IQ R Q + +YR
Sbjct  674  RIEKQLKRRFAIGSQVSEHSIIKDFT-KQKYPEHAIHKVLQLMLRRGEIQHRMQRKVLYR  732
```

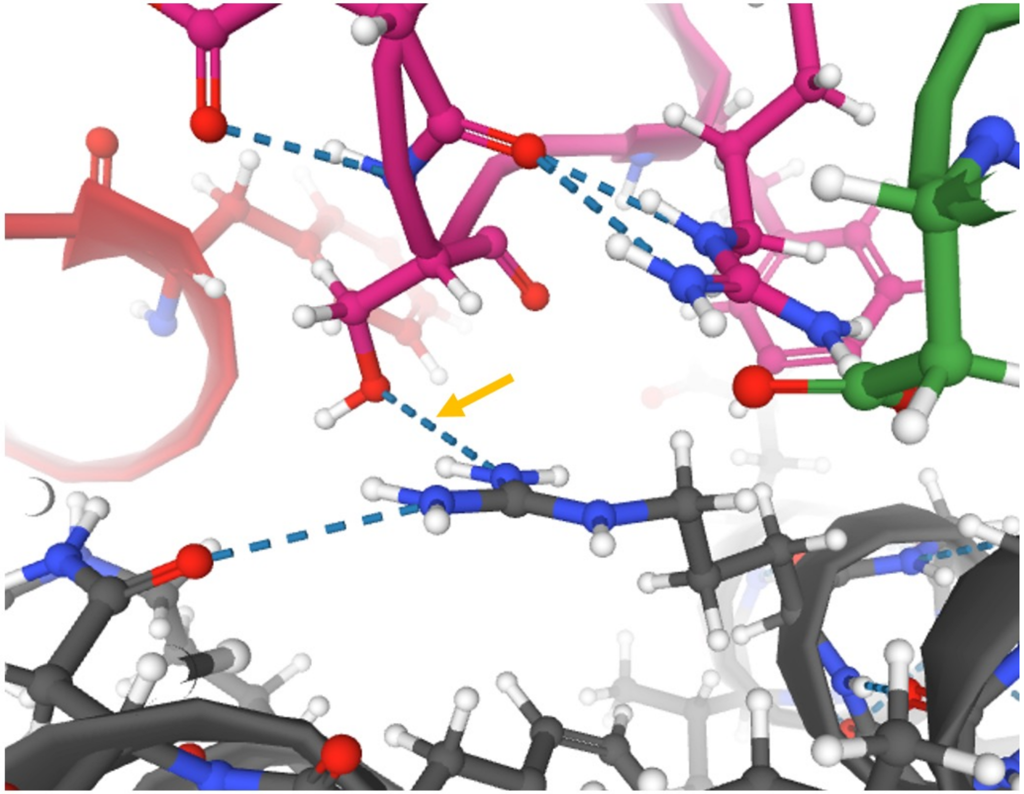

Supplementary figure 2E

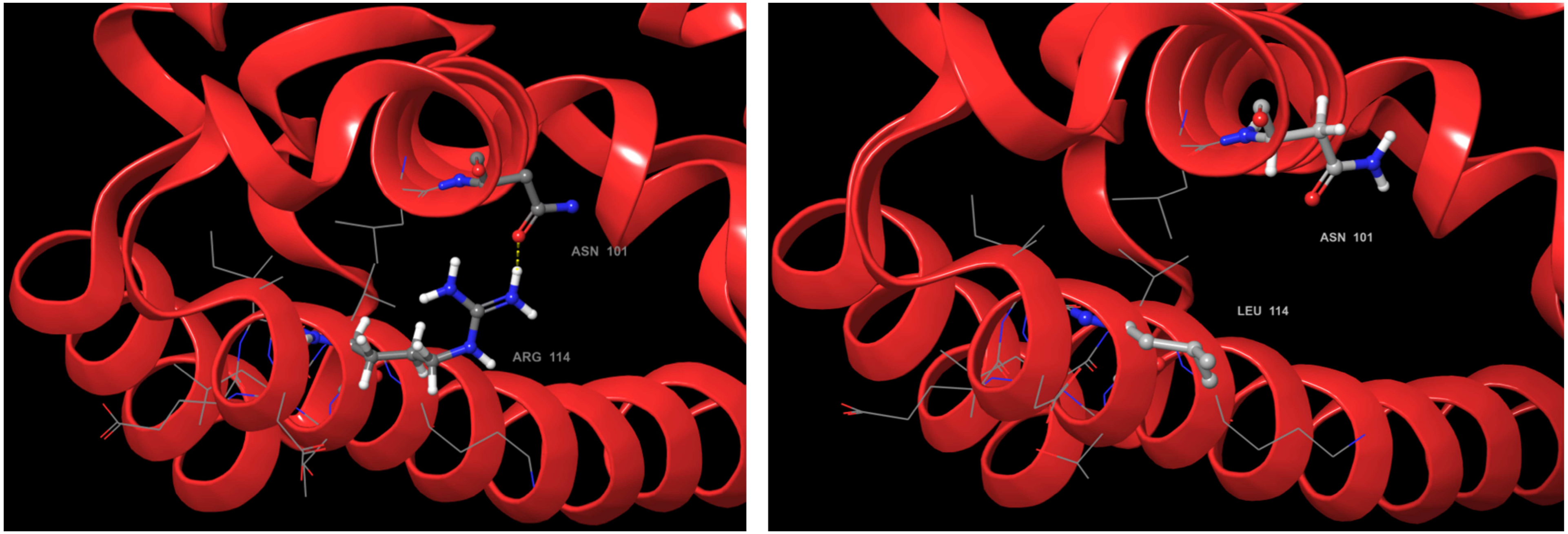

Supplementary figure 2F

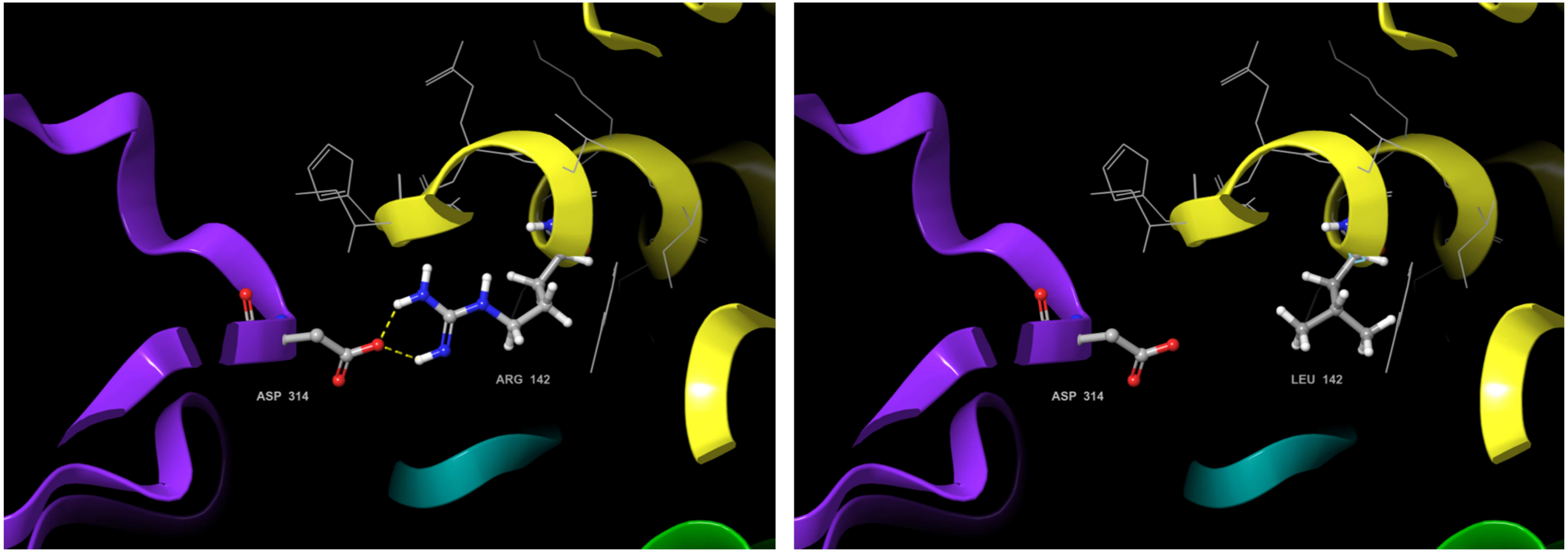

Supplementary figure 2G

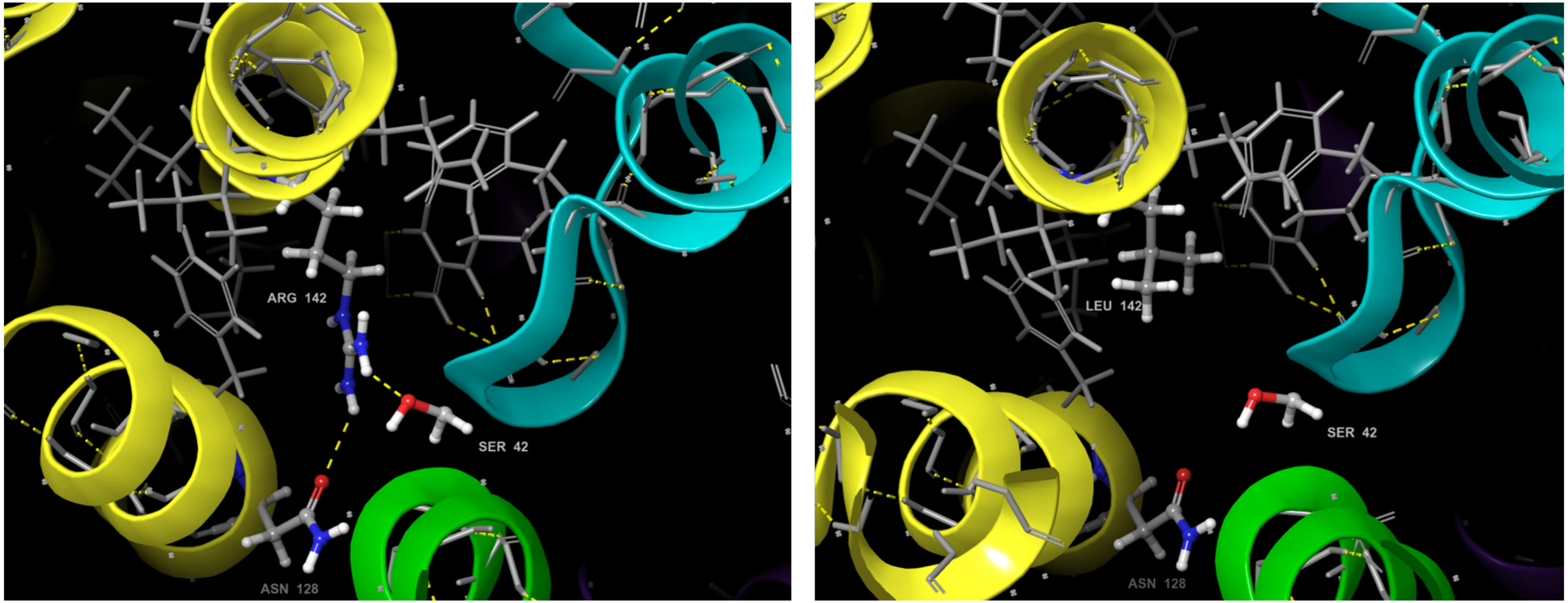

Supplement: Supplementary data [file jmedgenet-2020-107572supp005.pdf]
