# Supplementary material for: Biallelic GINS2 variant p.(Arg114Leu) causes Meier-Gorlin syndrome with craniosynostosis
Source: J Med Genet. 2021 Aug 5;59(8):776–80. doi: 10.1136/jmedgenet-2020-107572 (PMC9340002; doi:10.1136/jmedgenet-2020-107572)
Supplement: Supplementary data [file jmedgenet-2020-107572supp006.pdf]

Supplementary table 3. Clinical features of individuals with MGORS or immunodeficiency with genetic defects in components and regulators of the pre-replication and pre-initiation complexes involved in DNA replication

Gene	*GINS2*	*ORC1*	*ORC4*	*ORC6*	*CDT1*	*CDC6*	*GMNN*	*DONSON*	*CDC45*	*MCM5*	*Total*	*MCM4*	*GINS1*
Phenotype	*MGORS*	*MGORS*	*MGORS*	*MGORS*	*MGORS*	*MGORS*	*MGORS*	*MGORS*	*MGORS*	*MGORS*	*MGORS*	*Immuno-deficiency 54*	*Immuno-deficiency 55*
References (PMID)	Present report	21358633, 21358632, 21358631, 11477602	21358632, 21358631, 11477602	21358632, 25691413	21358632, 21358631, 11477602	21358632	26637980	31784481	27374770	28198391		22499342, 18430777, 22354167, 22354170	28414293
N (number of TOP)	1 (0)	10 (1)	7 (0)	5 (2)	8 (0)	1 (0)	3 (0)	4 (0)	15 (2)	1 (0)	55 (5)	21 (0)	5 (0)
Gender	F	5M, 5F	1M, 6F	4M, 1F	2M, 6F	M	1M, 2F	3M, 1F	7M, 8F	M	25M, 30F	10M, 11F	1M, 5F
Age at last examination	7y	17w GA - 47y	5y - 23y	20w GA - 15y 5m	4y 4m - 17y 6m	7y	34m - 17y	7y - 29y 10m	22w GA - 28y	4y 8m	17w GA - 47y	0.5y - 17.9y	18m - 29y
**Growth**													
*Intrauterine growth restriction**	+	+ (8/9)	+ (7/7)	+ (4/4)	+ (7/8)	+ (single case)	+ (2/3)	+ (4/4)	+ (10/15)	+ (single case)	+ (45/53)	+ (19/20)	+ (5/5)
*Short stature***	+	+ (9/9)	+ (6/7)	+ (3/3)	+ (6/8)	+	+ (3/3)	+ (4/4)	+ (10/13)	+	+ (44/50)	+ (18/21)	+ (4/5)
*Microcephaly****	+	+ (8/8)	+ (4/6)	+ (4/4)	+ (3/8)	+	+ (2/3)	+ (3/4)	+ (14/15)	-	+ (40/51)	+ (8/9)	NR
**Craniofacial ^**													
Microtia / small ears	+	+ (9/10)	+ (7/7)	+ (3/3)	+ (8/8)	+	+ (3/3)	+ (4/4)	+ (13/14)	+	+ (50/52)	NR	NR
Conductive hearing loss	-	+ (1/1)	+ (1/3)	NR	NR	NR	+ (1/3)	+ (2/2)	+ (2/3)	NR	+ (7/13)	NR	NR
Cleft palate	-	- (0/1)	NR	NR	NR	+	+ (1/1)	+ (1/1)	+ (2/2)	NR	+ (5/7)	NR	NR
Microstomia	+	+ (4/6)	+ (4/4)	+ (1/3)	+ (2/7)	-	+ (3/3)	NR	+ (5/8)	+	+ (21/34)	NR	NR
Full lips	+	+ (7/8)	+ (2/4)	+ (3/3)	+ (7/8)	+	+ (3/3)	+ (1/1)	+ (6/7)	+	+ (32/37)	NR	NR
Retro-/micrognathia	+	+ (5/8)	+ (4/4)	+ (5/5)	+ (7/8)	+	+ (3/3)	+ (1/1)	+ (8/8)	+	+ (36/40)	NR	NR
**Cardiovascular**													
Congenital heart defect	+	NR	+ (1/1)	- (0/1)	+ (1/1)	NR	NR	NR	+ (5/5)	NR	+ (8/9)	+ (1/2)	NR
**Respiratory**													
Congenital pulmonary emphysema	-	+ (3/4)	+ (1/4)	- (0/4)	+ (4/7)	- (0/1)	+ (1/3)	NR	NR	NR	+ (9/24)	NR	NR

Laryngo-/tracheo-/bronchomalacia	-	+ (1/1)	+ (2/2)	NR	NR	NR	+ (1/3)	NR	NR	NR	+ (4/7)	NR	NR
**Gastrointestinal**													
Feeding problems	+	+ (3/3)	+ (7/7)	+ (1/2)	+ (3/5)	+	+ (3/3)	NR	NR	-	+ (19/23)	NR	NR
Gastroesophageal reflux	+	+ (2/2)	+ (5/5)	NR	+ (1/1)	+	+ (1/3)	NR	NR	NR	+ (11/13)	+ (1/1)	NR
**Mammary glands**													
Postpubertal mammary hypo-/aplasia (female)	na	+ (2/2)	+ (5/5)	+ (1/1)	+ (3/3)	na	NR	NR	+ (1/1)	na	+ (12/12)	NR	NR
**Genitourinary ⊤**													
Hypoplastic labia minora/majora (female)	NR	NR	+ (3/3)	NR	+ (1/1)	na	+ (1/1)	NR	NR	na	+ (5/5)	NR	NR
Micropenis (male)	na	+ (1/3)	NR	+ (1/3)	- (0/2)	+	NR	NR	+ (1/1)	NR	+ (4/10)	NR	NR
Cryptorchidism (male)	na	+ (2/3)	NR	+ (2/2)	+ (1/2)	+	+ (1/1)	NR	+ (1/1)	+	+ (9/11)	NR	NR
**Skeletal**													
Craniosynostosis	+	+ (1/1)	NR	NR	NR	NR	NR	NR	+ (12/13)	NR	+ (14/15)	NR	NR
Delayed bone age	+	+ (3/3)	+ (1/3)	+ (2/3)	+ (3/4)	+	+ (2/2)	+ (1/1)	NR	NR	+ (14/18)	+ (5/5)	NR
Patellar aplasia/hypoplasia	+	+ (4/7)	+ (7/7)	+ (4/4)	+ (8/8)	+	+ (3/3)	+ (4/4)	+ (8/11)	+	+ (41/47)	NR	NR
**Neurologic**													
Development delay / Intellectual disability	-	+ (2/8)	- (0/7)	- (0/3)	- (0/8)	-	+ (2/3)	+ (3/4)	+ (3/12)	-	+ (10/48)	+ (5/6)	NR
**Immunologic**													
Recurrent infections	+	+ (1/1)	NR	NR	+ (1/1)	NR	NR	NR	NR	+	+ (4/4)	+ (9/16)	+ (5/5)
NK cell deficiency	-	NR	NR	NR	NR	NR	NR	NR	NR	-	- (0/2)	+ (16/16)	+ (5/5)
Neutropenia	-	NR	NR	NR	NR	NR	NR	NR	NR	-	- (0/2)	+ (3/3)	+ (5/5)
**Endocrinologic**													
Adrenal insufficiency	NR	NR	NR	NR	NR	NR	NR	NR	NR	NR	NR	+ (20/21)	NR

na, not applicable. NR, not reported. M, male. F, female. m, months. Y, years-old. GA, gestational age. w, weeks *Abnormal restriction of fetal growth with fetal weight below the tenth percentile for gestational age. **Height more than 2 standard deviations below the mean for age and gender. ***Head circumference more than 2 standard deviations below the mean for age and gender. ^Thin eyebrows were reported in all 15 individuals with biallelic *CDC45* variants. ⊤Anal abnormalities were reported in 7/7 individuals with biallelic *CDC45* variants. NB. This case series review was based on the data of 80 individuals previously reported in the literature. In retrospective studies, which rely on data from existing records and mostly not collected for research purposes, clinical information may be missing (e.g. individuals may have developed a feature that was not reported, or they may still be at risk and not have developed the clinical feature at the time of the study). Thus, "Attention should be paid to the missing data in the analysis of the resulting data, as missing data can reduce the representativeness of the samples and can produce biased estimates, leading to invalid conclusions (PMID 23741561; PMID 32847800)." A detailed molecular and clinical characterization of each individual included in this review is available in Supplementary table 3.
